# Draft genome sequences of 21 *Bacillus* sp. isolates from raw bovine milk

**DOI:** 10.1128/mra.00569-24

**Published:** 2024-08-20

**Authors:** Gauri Khullar, Zhangbin Cai, Bridget O'Brien, Jinha Suh, Jennifer Ronholm

**Affiliations:** 1 Faculty of Agricultural and Environmental Sciences, Macdonald Campus, McGill University, Montréal, Québec, Canada; 2 Department of Food Technology, Faculty of Science, Chulalongkorn University, Patumwan, Bangkok, Thailand; 3 Mastitis Network, Saint-Hyacinthe, Québec, Canada; 4 Regroupement FRQNT Op+Lait, Saint-Hyacinthe, Québec, Canada; Department of Biological Sciences, Wellesley College, Wellesley, Massachusetts, USA

**Keywords:** *Bacillus*, milk, probiotic, healthy, bovine

## Abstract

Members of the *Bacillus* genus are commonly used as probiotics in livestock production. We isolated several *Bacillus* strains from healthy dairy cattle. The role of these strains in mammary health is of interest. Here, we present 21 draft genome assemblies and annotations of *Bacillus* sp. isolated from fresh raw milk.

## ANNOUNCEMENT

Members from the *Bacillus* genus, such as *Bacillus subtilis, B. coagulans*, *B. cereus*, and *B. licheniformis*, are commonly used as probiotics in livestock production (1–3). *Bacillus* probiotics can improve digestion, increase nutrient absorption, and reduce gas emissions (4–6). Some may also prevent bacterial infections. Members of the *Bacillus* genus can produce lipopeptides, surfactins, bacteriocins, and organic acids, each of which has antimicrobial activity (1).

Intramammary infections (IMIs) are common in dairy cattle (7). Evidence indicates that *Bacillus* sp. may have antagonistic effects against IMI pathogens and may be used to prevent IMIs. For example, feeding cattle *B. subtilis* decreased the incidence of IMIs (8). *Bacillus* sp. isolated from healthy cows can inhibit the growth of several gram-positive IMI pathogens in co-culture (9), and exopolysaccharides synthesized by *Bacillus velezensis* have anti-biofilm activity against *Staphylococcus aureus* (10). Although cross-species use of *Bacillus* probiotics is common (11), it is understood that host colonization is more successful if the probiotic strain was isolated from the species and host niche the probiotic aims to colonize. To develop bovine anti-IMI probiotics, we collected 21 *Bacillus* isolates from healthy Holstein dairy cows. Here, we present draft genome sequences of these isolates.

Raw milk was collected directly from a single healthy cow on the Macdonald campus farm of McGill University (45.41168, -73.94355). The milk was transferred to an Eppendorf tube and was centrifuged at 3,800 × *g* for 20 min (12). The supernatant was discarded, and the pellet was spread on modified plate count agar (Hardy Diagnostics, USA). Plates were incubated overnight at 37°C. Isolated colonies were differentiated based on phenotypic properties. Unique colonies were grown in Brain Heart Infusion (BHI) broth for 48 h at 37°C. Aliquots of 1 mL of bacterial culture were preserved by adding 15% glycerol (vol/vol) and were stored at −80°C. Each isolate was tentatively identified by Sanger Sequencing of the full-length 16S rRNA gene using the 8F (5′-AGAGTTTGATCCTGGCTCAG-3′) and 1492R (5′-ACCTTGTTACGACTT-3′) primers.

Each −80°C stock solution was streaked onto a BHI agar plate and was incubated overnight at 37°C. A single colony was inoculated into 20 mL of BHI broth and was grown overnight at 37°C and 200 rpm. The DNeasy UltraClean Microbial Kit DNA was used to extract bacterial DNA from liquid cultures (Qiagen, USA). The Quant-IT dsDNA High-Sensitivity assay (Invitrogen, USA) was used to assess the final DNA quantity. Sequencing libraries were made using the Nextera DNA Flex Prep kit and were sequenced using the MiSeq benchtop sequencer and the MiSeq Reagent Kit v3 (2 × 300 bp) (Illumina, USA). All software used default parameters unless otherwise noted. Reads were assembled using ProkaryoteAssembly (v. 0.1.6) (https://github.com/BFSSI-Bioinformatics-Lab/ProkaryoteAssembly), filtering low-quality sequences with a Q-score <20. Following assembly, contigs with fewer than 1,000 bp were removed (Table 1). Quality of final assemblies was assessed with QualiMap (v. 2.2.2) (13). Gene annotations and predictions were performed using NCBI Prokaryotic Genome Annotation Pipeline (v. 6.7) (14). The annotation and functional assignment were validated using Rapid Annotations Subsystems Technology (RAST) kit (15). The completeness of the genome assemblies was assessed using Benchmarking Universal Single-Copy Orthologs (BUSCO v5) (16).

**TABLE 1:** Genome quality and prediction of *Bacillus* sp. isolated from bovine milk in Canada^*a*^

Isolate identification no.	Species	Assembly accession no.	BioSample accession no.	SRA accession no.	GenBank accession no.	Coverage (X)	No. of contigs	Draft genome size (bp)	GC content (%)	No. of CDS	No. ofRNAs	N50 (bp)
1	*B. licheniformis*	GCF_039704985.1	SAMN39861007	SRR28833364	JBDICK000000000	23.9734	53	4075575	46.189556	4439	69	161839
7	*B. licheniformis*	GCF_039704965.1	SAMN39861008	SRR28833363	JBDICJ000000000	56.1865	27	4075098	46.189	4432	68	402185
23b	*B. licheniformis*	GCF_039704945.1	SAMN39861009	SRR28833352	JBDICI000000000	73.6856	27	4121131	46.152622	4502	69	298889
21_C	*B. licheniformis*	GCF_039704925.1	SAMN39861010	SRR28833348	JBDICH000000000	31.8968	31	4410271	45.68565	4925	81	287648
27	*B. licheniformis*	GCF_039704865.1	SAMN39861011	SRR28833347	JBDICG000000000	18.6028	107	4273296	45.819504	4793	80	66976
33	*B. licheniformis*	GCF_039704875.1	SAMN39861012	SRR28833346	JBDICF000000000	62.6018	28	4410102	45.686153	4937	79	346695
35	*B. licheniformis*	GCF_039704845.1	SAMN39861013	SRR28833345	JBDICE000000000	57.1802	40	4429233	45.64856	4957	71	296898
39	*B. licheniformis*	GCF_039704855.1	SAMN39861014	SRR28833344	JBDICD000000000	65.0995	29	4283372	45.843018	4726	71	296898
2	*B. licheniformis*	GCA_039704785.1	SAMN39861015	SRR28833343	JBDICC000000000	38.0009	32	4423964	45.74099	4944	70	284038
8	*B. licheniformis*	GCF_039704805.1	SAMN39861016	SRR28833342	JBDICB000000000	76.2563	31	4425022	45.74045	4934	70	296898
11	*B. licheniformis*	GCF_039704745.1	SAMN39861017	SRR28833362	JBDICA000000000	28.0249	35	4285446	45.831657	4731	81	296898
17a	*B. licheniformis*	GCF_039704765.1	SAMN39861018	SRR28833361	JBDIBZ000000000	116.5275	31	4361801	45.669575	4893	72	424554
5	*B. licheniformis*	GCF_039704755.1	SAMN39861019	SRR28833360	JBDIBY000000000	37.4583	32	4345927	45.808407	4811	67	228812
28	*B. licheniformis*	GCF_039704725.1	SAMN39861020	SRR28833359	JBDIBX000000000	65.022	29	4284751	45.8404	4725	71	296898
32	*B. licheniformis*	GCF_039704565.1	SAMN39861021	SRR28833358	JBDIBW000000000	25.8095	29	4396779	45.70391	4920	69	296898
38	*B. licheniformis*	GCF_039704625.1	SAMN39861022	SRR28833357	JBDIBV000000000	16.7513	196	4382965	45.650055	5040	69	46449
4	*B. pumilus*	GCF_039704575.1	SAMN39861023	SRR28833356	JBDIBU000000000	47.1156	16	3685821	41.56838	3841	62	932876
7_C	*B. pumilus*	GCF_039704555.1	SAMN39861024	SRR28833355	JBDIBT000000000	43.1238	18	3684661	41.562927	3839	62	932849
1_C	*B. safensis*	GCF_039704545.1	SAMN39861025	SRR28833354	JBDIBS000000000	22.8362	26	3638373	41.65884	3793	58	256236
3	*B. aerius*	GCF_039581685.1	SAMN41074328	SRR28833353	JBCPSH000000000	81.0985	16	3718211	41.24785	3869	64	540547
21	*B. subtilis*	GCF_039581705.1	SAMN41074331	SRR28833349	JBCPSF000000000	104.0174	9	4145080	43.58461	4347	70	2210931

^*a*^ CDS, coding sequence; RNAs, sum of rRNA and tRNA counts.

## Data Availability

The raw reads have been submitted to SRA, and the genome sequences have been deposited in DDBJ/ENA/GenBank under the BioProject accession numbers PRJNA1074428 and PRJNA1104282, as provided in Table 1.
